# Whisker-Mediated Touch System in Rodents: From Neuron to Behavior

**DOI:** 10.3389/fnsys.2019.00040

**Published:** 2019-08-21

**Authors:** Mehdi Adibi

**Affiliations:** ^1^School of Psychology, University of New South Wales, Sydney, NSW, Australia; ^2^Tactile Perception and Learning Lab, International School for Advanced Studies (SISSA), Trieste, Italy; ^3^Padua Neuroscience Center, University of Padua, Padua, Italy

**Keywords:** rodents, whisker system, vibrissae, vibrissal system, somatosensory, barrel field, thalamic barreloids

## Abstract

A key question in systems neuroscience is to identify how sensory stimuli are represented in neuronal activity, and how the activity of sensory neurons in turn is “read out” by downstream neurons and give rise to behavior. The choice of a proper model system to address these questions, is therefore a crucial step. Over the past decade, the increasingly powerful array of experimental approaches that has become available in non-primate models (e.g., optogenetics and two-photon imaging) has spurred a renewed interest for the use of rodent models in systems neuroscience research. Here, I introduce the rodent whisker-mediated touch system as a structurally well-established and well-organized model system which, despite its simplicity, gives rise to complex behaviors. This system serves as a behaviorally efficient model system; known as nocturnal animals, along with their olfaction, rodents rely on their whisker-mediated touch system to collect information about their surrounding environment. Moreover, this system represents a well-studied circuitry with a somatotopic organization. At every stage of processing, one can identify anatomical and functional topographic maps of whiskers; “barrelettes” in the brainstem nuclei, “barreloids” in the sensory thalamus, and “barrels” in the cortex. This article provides a brief review on the basic anatomy and function of the whisker system in rodents.

## 1. Introduction

A fundamental goal of systems neuroscience is to identify how sensory stimuli are represented in neuronal activity, and how the activity of sensory neurons is “read out” by downstream neuronal structures to generate behavior. Researchers dissect this goal into the following questions:

1. What elemental features of sensory stimuli are encoded in the neuronal activity of sensory neurons?
2. How is each elemental feature represented in the activity of sensory neurons?
3. How do the downstream neuronal areas decode the activity of sensory neurons?
4. How does spatial and temporal context affect the efficiency with which single neurons and neuronal ensembles encode sensory stimuli?
5. How does the activity of neurons give rise to perception and ultimately behavior?

Over the past decade, the increasingly powerful array of experimental approaches such as optogenetics and two-photon imaging which has become available in non-primate models, particularly in rodents, has spurred a renewed interest for the use of rodents in neuroscience research. The aim of this article is to introduce the rodent whisker-mediated touch system as a model system suitable for investigating the fundamental questions in systems neuroscience. This model serves as an anatomically well-established and behaviorally efficient system; as nocturnal animals, rodents rely on their whisker-mediated touch system to collect information about their surrounding environment. Moreover, this system represents a well-studied circuitry with an elegant structural organization. At every stage of processing, one can identify anatomical and functional topographic maps of whiskers. These clusters are referred to as “barrelettes” in the brainstem nuclei, “barreloids” in the thalamus, and “barrels” in the cortex. Mapping studies have revealed that whisker-related areas occupy a relatively large proportion of neural tissue at trigeminal medullar level (28%) (Nord, 1967), at the level of thalamic sensory nuclei (27%) (Emmers, 1965), and at the cortical level (20%) (Welker, 1971).

In the following sections, I first provide a brief introduction to the basic anatomy and then the function of the whisker system in rodents.

## 2. The Whisker-Mediated Touch System

### 2.1. Vibrissae and Follicles

**Figure 1:**
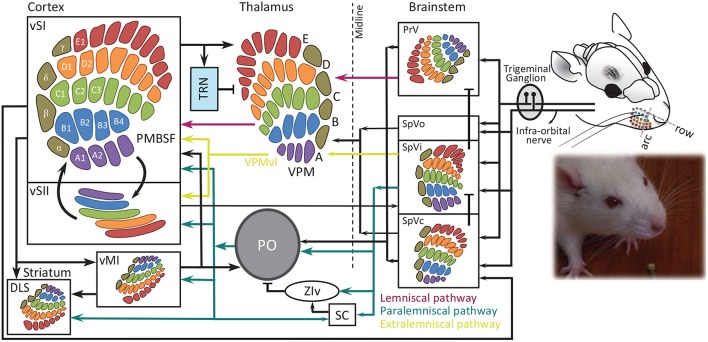
Schematic representation of whisker-barrel system. Each whisker is identified by a unique letter-number combination corresponding to its row (A to E from dorsal to ventral) and arc (identified by numbers 1, 2 and etcetera from caudal to rostral), with α, β, γ, and δ straddlers between rows. Colors indicate rows. Barrel, barreloid and barrelets are redrawn from Durham and Woolsey (1984). PMBSF, posterior-medial barrel sub-field; PO, posterior thalamic nucleus; PrV, principal trigeminal nucleus; SC, superior colliculus; SpVi, spinal trigeminal nuclei pars interpolaris; SpVo, pars oralis; SpVc, pars caudalis; TRN, thalamic reticular nucleus; VPM, ventro-posterior medial nucleus; vMI, vibrissal primary motor cortex; vSI, vibrissal primary somatosensory cortex; vSII, secondary somatosensory cortex with the somatotopic map from Benison et al. (2007); DLS, dorsolateral striatum; ZIv, ventral zona inserta. The evidence for somatotopic map in vM1 is provided in Ferezou et al. (2007) and Sreenivasan et al. (2016).

Rat vibrissae, or whiskers, form a grid-wise layout on either side of the snout. The main distinction of the vibrissae from ordinary hairs is their large follicles which contain dense nerve terminals and sensory receptors. As mechanical transducers, the vibrissae mediate the transfer of the touch signal into these receptors. The vibrissae are categorized into two classes: (i) the micro-vibrissae, which are short and thin hairs around the nose tip, and (ii) macro-vibrissae, which are the long stiff mystacial hairs caudal to micro-vibrissae on the whisker pad (Brecht et al., 1997). Macro-vibrissae consist of four follicles in rows A and B, seven to nine follicles in row C, D and E, and four straddlers (α, β, γ, δ) straddling between rows caudal to the mystacial pad (see Figure 1).

These two classes of vibrissae are believed to be functionally distinct (Vincent, 1912; Brecht et al., 1997); the macro-vibrissae transmit spatial information such as localization in space, as they sweep the environment by intrinsic muscles. However micro-vibrissae are considered to be involved in acquisition of detailed tactile information for object and texture recognition. Nevertheless, there is evidence from behavioral studies demonstrating that rodents are able to perform texture and vibration discrimination tasks using their macro-vibrissae (Carvell and Simons, 1990; Krupa et al., 2001; von Heimendahl et al., 2007; Adibi and Arabzadeh, 2011; Morita et al., 2011; Adibi et al., 2012).

The nerve terminals and mechanoreceptors around the vibrissa shaft are of various types, morphologies and distributions (Melaragno and Montagna, 1953) including Merkel cell-neurite complexes, lanceolate receptors, Ruffini corpuscles—sometimes referred to as reticular endings—and free nerve endings (Renehan and Munger, 1986; Rice et al., 1986; Ebara et al., 2002). Different receptors show different tuning properties and sensitivity to a variety of tactile stimulus parameters such as amplitude, frequency, duration, velocity, acceleration and direction of whisker deflections/motion (Fitzgerald, 1940; Kerr and Lysak, 1964; Zucker and Welker, 1969; Hahn, 1971; Pubols et al., 1973; Dykes, 1975; Gibson and Welker, 1983a,b; Lichtenstein et al., 1990). These receptors also exhibit different profiles of adaptation. Merkel cells are the most prominent mechanoreceptors. These receptors adapt slowly to sustained whisker deflections, whereas lanceolate receptors and simple corpuscles are rapidly-adapting (Iggo and Muir, 1969; Zucker and Welker, 1969; Munger et al., 1971; Gottschaldt et al., 1973; Pubols et al., 1973; Dykes, 1975).

Each follicle is innervated by 150–200 myelinated and 100 unmyelinated distal axons of trigeminal ganglion neurons (Lee and Woolsey, 1975; Waite and Cragg, 1982; Renehan and Munger, 1986; Rice et al., 1986, 1997; Henderson and Jacquin, 1995). These axons arborize around the hair shaft, sensing movements in different directions.

### 2.2. Whisking

Whisking is the rhythmic cyclic vibrissae sweeping action, consisting of repetitive forward (protraction) and backward (retraction) movements at an average frequency of about 8 Hz (Welker, 1964; Wineski, 1983; Carvell et al., 1991). Whisking is often synchronous to respiratory, head, and nose movements, suggesting coordination of activity among many muscle groups (Welker, 1964; Cao et al., 2012). Berg and Kleinfeld (2003) observed two different patterns of whisking; the first pattern, referred to as the exploratory whisking, consists of wide-angle sweeps with a frequency range of 1 to 5 Hz in bouts of 1 to 10 s. The whisking frequency within a bout remains remarkably constant, while it changes between bouts (Vincent, 1912; Welker, 1964; Wineski, 1983; Carvell and Simons, 1990; Carvell et al., 1991). The second pattern of whisking consists of small-amplitude high-frequency (ranging from 15 to 25 Hz) sweeps for a period of 0.5 to 1 s while whiskers are thrust forward in a dense pattern (Carvell and Simons, 1990, 1995; Berg and Kleinfeld, 2003). This pattern resembles the dense focalized arrangement of photoreceptors in the retina fovea, and is therefore referred to as “foveal whisking”. Movement of the follicle is controlled by the facial motor nerve. Macro-vibrissae are moved by two sets of striated musculatures (Dörfl, 1982; Wineski, 1983, 1985); the intrinsic and extrinsic muscles. Intrinsic muscles lack a bony attachment and have their origin and insertions in the skin (Dörfl, 1982). They are associated with individual whiskers and generate the forward whisker motion (protraction) by pulling the base of the follicle backwards (Carvell et al., 1991). Extrinsic muscles are located superficially in the mystacial pad with no direct connection with follicles. They move all whiskers together (Wineski, 1983, 1985; Dörfl, 1985; Carvell et al., 1991). On the basis of anatomical observations, Dörfl (1982, 1985) and Wineski (1985) concluded that mystacial pad muscles move the whiskers forward (protraction), whereas backward motion (retraction) is mainly a result of the elastic properties of the facial tissue, and is therefore passive. A more recent finding, however, demonstrated that retraction is under the active muscular control as well (Berg and Kleinfeld, 2003).

Whisking is controlled by a neuronal oscillator located in the vibrissa-related region of intermediate reticular formation of the medulla (vIRt) (Moore et al., 2013, 2014; Deschênes et al., 2016). This region includes facial premotor neurons and neurons that their spiking activity is either in phase or in anti-phase with whisking protraction. Selective lesions in vIRt abolish whisking on the side of the lesion, and activation of the vIRt by iontophoretic injection of kainic acid (KA) induces long episodes of whisking under light ketamine anesthesia (Moore et al., 2014). Glycinergic/GABAergic neurons in vIRt rhythmically inhibit vibrissa facial motoneurons innervating the intrinsic muscles (Deschênes et al., 2016), suggesting that rhythmic whisking is driven by inhibition. During whisking, the intrinsic muscles protracting individual whiskers follow the whisking oscillation, while extrinsic muscles that move the mystacial pad follow the breathing rhythm. Both rhythms are phase-locked during sniffing (rapid rhythmic breathing) (Deschênes et al., 2012; Kleinfeld et al., 2014). This is compatible with the unidirectional connections from the pre-Bötzinger complex—the inspiratory oscillator for respiration located in medulla adjacent to IRt (Feldman and Kam, 2015)—to vIRt, revealing the contribution of pre-Bötzinger complex to the mystacial pad control by driving the extrinsic muscles together with the potential contribution of putative parafacial neurons that receive their input from pre-Bötzinger complex (Deschênes et al., 2016). There are no bilateral vIRt to vIRt connections. Thus, the bilateral synchronization of whisking is mediated by the medullary commissural fibers connecting the left and right pre-Bötzinger complexes (Deschênes et al., 2016).

Whiskers on the right and left sides can move asymmetrically and asynchronously (Knutsen et al., 2006; Towal and Hartmann, 2008). Additionally, rostral and caudal whiskers on a single side of the snout can sometimes move independently. Recently, using a three-dimensional model of the vibrissal array, Huet and Hartmann (2014) quantified the search space during whisking and protraction. According to their calculations, the parabolic intrinsic curvature of the whiskers increases the volume of the search space by over 40% compared to that of the straight whiskers, while the elevation—whisker's angle relative to the horizontal plane—and torsion—torsional rotation of a whisker about its own axis—had modest effect on the search space. Elevation and torsion, however, affect the trajectory of the whisker tips. Dynamics of whisker movement reveal a rodent's expectations about the environment (Mitchinson et al., 2007; Grant et al., 2009). During locomotion, direction and speed of running are coupled with average whisker position (Towal and Hartmann, 2006, 2008; Mitchinson et al., 2011; Sofroniew et al., 2014). The fine-scale kinematics of the whisking motion in freely moving rodents, however, is difficult to characterize. Machine learning techniques such as deep learning (Hong et al., 2015), visually enhanced whiskers for tracking using florescent dyes (Rigosa et al., 2017) and precise controlled locomotion in virtual reality for head-fixed animal (Sofroniew et al., 2014) are promising future approaches for high precision characterization of whisker motion kinematics during locomotion.

### 2.3. Trigeminal Ganglion

Trigeminal ganglion (also called semilunar ganglion) consists of the cell bodies of pseudo-unipolar neurons with their proximal axons innervating the ipsilateral brainstem trigeminal complex (BTC) (Vincent, 1913; Ma and Woolsey, 1984) and their distal axons innervating the vibrissae follicles. Each ganglion cell innervates only one whisker follicle (Fitzgerald, 1940; Zucker and Welker, 1969; Dykes, 1975; Gibson and Welker, 1983a; Rice et al., 1986; Lichtenstein et al., 1990). The trigeminal ganglion is somatotopically organized with caudal arcs represented dorsally, and dorsal rows represented medially (Zucker and Welker, 1969; Lichtenstein et al., 1990). Early studies indicate that a great majority of the ganglion cells are slowly adapting (Fitzgerald, 1940; Kerr and Lysak, 1964; Zucker and Welker, 1969; Lichtenstein et al., 1990, but see Pubols et al., 1973; Gibson and Welker, 1983b). The rapidly adapting ganglion cells have generally higher velocity thresholds (Zucker and Welker, 1969; Lichtenstein et al., 1990). Different trigeminal ganglion units show various tuning properties, with evidence suggesting sensitivity to the following parameters: amplitude, frequency, duration, velocity, acceleration and direction of whisker deflections/motion (Fitzgerald, 1940; Kerr and Lysak, 1964; Zucker and Welker, 1969; Hahn, 1971; Pubols et al., 1973; Dykes, 1975; Gibson and Welker, 1983a,b; Lichtenstein et al., 1990). These neurons are highly sensitive to whisker deflection with over 50% of units responding to <1° of whisker deflection (Gibson and Welker, 1983a). The spontaneous activity of these units is considered to be zero (Zucker and Welker, 1969; Gibson and Welker, 1983a; Lichtenstein et al., 1990), and any discharge is potentially attributed to the high sensitivity of the units to tiny movements such as pneumatic vibrations, mechanical hysteresis of hair shaft, or tissue damage caused by microelectrode penetration (Gibson and Welker, 1983a).

### 2.4. Brainstem Trigeminal Complex (BTC)

Traditionally, the whisker-recipient trigeminal complex in the brainstem is subdivided into the principal sensory nucleus (PrV) and the spinal nucleus (SpV). The latter is further subdivided rostro-caudally into 3 sub-nuclei: oralis (SpVo), interpolaris (SpVi) and caudalis (SpVc) (Arvidsson, 1982; Ma and Woolsey, 1984). Trigeminal nuclei neurons receive inputs from trigeminal ganglion cells and form discrete aggregated neuronal clusters—called barrelettes—in each nucleus except for SpVo (Erzurumlu and Killackey, 1980; Durham and Woolsey, 1984; Bates and Killackey, 1985; Chiaia et al., 1991; Ma, 1991; Jacquin et al., 1993). Brainstem barrelettes preserve the somatotopic organization of whiskers on the mystacial pad (Belford and Killackey, 1979; Hayashi, 1980; Arvidsson, 1982). Each barrelette is about 55 μm in diameter and 1.2 mm long along the rostro-caudal direction and contains 160–200 neurons (Timofeeva et al., 2003). The PrV and SpVi sub-nuclei provide the majority of the projections to the thalamus. Similar to first-order neurons in trigeminal ganglion, the more sensitive BTC units (with low velocity thresholds) were slowly adapting, whereas the less sensitive units (high velocity thresholds) were rapidly adapting.

A majority of PrV barrelette neurons have barrelette-bounded dendritic trees (Jacquin et al., 1993; Veinante and Deschênes, 1999). These neurons mainly project into single barreloids—neuronal aggregates representing individual whiskers—of the ventro-posterior medial nucleus (VPM) in the contralateral thalamus (Jacquin et al., 1988; Veinante and Deschênes, 1999). Other groups of neurons in PrV with large multipolar somata and expansive dendritic branches spread over multiple barrelettes (Jacquin et al., 1988; Jacquin and Rhoadesi, 1990; Veinante and Deschênes, 1999), and also respond to multiple whiskers. This population mainly projects into the posterior thalamic nucleus (POm) in thalamus, tectum, superior colliculus, zona incerta, the medial part of the medial geniculate nucleus (MGm), inferior olive and medial dorsal part of VPM (VPMdm) (Huerta et al., 1983; Bruce et al., 1987; Bennett-Clarke et al., 1992; Van Ham and Yeo, 1992; Williams et al., 1994; Veinante and Deschênes, 1999). The electrophysiological studies identified two broad classes of neurons in PrV; tonic neurons which represent a single whisker, and phasic units which are driven by single or multiple whiskers (Shipley, 1974; Veinante and Deschênes, 1999; Minnery and Simons, 2003; Minnery et al., 2003).

Neurons in SpVi spread their dendritic arbors into a broader area across multiple barrelettes, and thus respond to multiple whiskers (Woolston et al., 1982; Jacquin et al., 1986). These neurons project to different brain areas, such as ventrobasal complex (mainly ventro-lateral VPM, VPMvl), the zona incerta, superior colliculus, medial geniculate nucleus, cerebellum and spinal cord (Erzurumlu and Killackey, 1980; Huerta et al., 1983; Silverman and Kruger, 1985; Jacquin et al., 1989; Van Ham and Yeo, 1992; Williams et al., 1994). SpVc also projects to VPMvl similar to the thin axons of SpVi. SpVo sends a few axons only to POm (Veinante et al., 2000).

### 2.5. Thalamus

VPM, POm and the intralaminar thalamic nuclei form the major thalamic targets of second-order neurons of brainstem trigeminal complex (Williams et al., 1994; Diamond, 1995; Veinante and Deschênes, 1999). The vibrissae representation area in VPM is somatotopically organized into discrete finger-like structures, called barreloids (van der Loos, 1976). Barreloids are oblong cylinder-like structures, with a length of 500–900 μm and contain 250 to 300 neurons each (van der Loos, 1976; Saporta and Kruger, 1977; Land et al., 1995; Timofeeva et al., 2003; Oberlaender et al., 2012). The size of the barreloids is positively correlated with the length of whiskers (Haidarliu and Ahissar, 2001). Cells within a barreloid have receptive fields composed of one principal and several surrounding whiskers (Friedberg et al., 1999). POm is more homogeneous than VPM, with no barreloid-like structures. However, there is evidence that POm is organized topographically (Diamond et al., 1992; Alloway et al., 2003). Compared to VPM cells, the receptive field of POm neurons is larger (6–8 whiskers) (Diamond et al., 1992). Moreover, POm neurons show a weaker response to single whisker deflections than VPM neurons do, and unlike VPM neurons, POm neurons exhibit less preference to a particular principal whisker (Diamond et al., 1992). Instead, POm neurons are strongly driven by simultaneous disturbance of multiple whiskers.

Thalamic barreloids receive three main inputs:

1. an ascending excitatory input from the principal trigeminal nucleus (PrV),
2. an excitatory corticothalamic input from the barrel field in the primary somatosensory cortex (SI),
3. an inhibitory input from the thalamic reticular nucleus.

In all of these pathways, terminal fields of axons are mainly confined to the barreloid representing the corresponding principal whisker of their receptive field (Williams et al., 1994; Veinante and Deschênes, 1999; Desilets-Roy et al., 2002; Varga et al., 2002). The distal dendritic arbors of a proportion of VPM cells, however, spread in the surrounding barreloids, leading to a cross-whisker interaction (Varga et al., 2002). In contrast to the sensory-thalamic nuclei for other modalities, there are few, if any, dendrodendritic synapses and no local axon collaterals and inhibitory interneurons in rat VPM (Barbaresi et al., 1986; Harris, 1986).

Afferents of VPMdm neurons of thalamic barreloids arborize in the corresponding neuronal aggregates—barrels—in layer IV of primary somatosensory cortex and form a one-to-one connection between the VPM barreloids and cortical barrels (Herkenham, 1980; Jensen and Killackey, 1987; Chmielowska et al., 1989; Lu and Lin, 1993). Multi-barrel projections of VPM neurons have never been observed. However, some axonal innervations into septal regions surrounding the barrels were found. Thalamic reticular nucleus and the upper part of layer VI of barrel field in SI are innervated by collaterals of the ascending projections from VPM (Jones, 1975; Herkenham, 1980; Jensen and Killackey, 1987; Chmielowska et al., 1989; Lu and Lin, 1993). The VPMvl neurons do not directly project to the barrels. They receive presynaptic inputs from the caudal division of SpVi and branch their axons in the secondary somatosensory cortex (SII) as well as septal and dysgranular zone in SI (Pierret et al., 2000) and form the extralemniscal pathway (Yu et al., 2006). An additional ascending pathway parallel to lemniscal pathway originates from multi-whisker PrV neurons passing through the head of the thalamic barreloids (Urbain and Deschênes, 2007). The neurons in the head of barreloids have multi-whisker receptive fields, innervate layer 4 septa and receive corticothalamic feedback from layer 6 of vibrissal MI (Urbain and Deschênes, 2007; Furuta et al., 2009). Hence it suggests this pathway is involved in relaying information related to the phase of whisking.

POm projects to almost all sensory-motor areas of the neocortex, including the primary somatosensory, secondary somatosensory (SII), perirhinal, insular and motor cortices, and to a lesser extent to thalamic reticular nucleus (Deschênes et al., 1998). The laminar distribution of the terminal fields of POm projection to cortex are mainly to layers Va and I (Deschênes et al., 1998). Similarly, POm axon terminals in SI are distributed from upper layer V to layer I of the dysgranular zone and interbarrel septa, as well as in layers V and I of the barrels (Herkenham, 1986; Koralek et al., 1988; Lu and Lin, 1993; Deschênes et al., 1998).

The thalamic reticular nucleus (TRN) with ventrobasal thalamic nuclei forms an inhibitory feedback loop which is believed to play role in thalamic spindling (Steriade et al., 1985; Fuentealba and Steriade, 2005), sleep-related thalamocortical oscillations (Steriade et al., 1993; Pinault, 2004; Fernández et al., 2018b), arousal (Steriade et al., 1986, 1993; Lewis et al., 2015), and selective attention (Skinner and Yingling, 1977; Crick, 1984). Optogenetic activation of TRN switches the thalamocortical firing pattern from tonic to bursting and enhances cortical spindles and delta waves (Halassa et al., 2011; Lewis et al., 2015). Neurons in the reticular nucleus receive vibrissae-related input from cortical Layer VI neurons in SI (Bourassa et al., 1995), collaterals from thalamocortical neurons in VPM and POm (Harris, 1987), as well as inputs from neighboring neurons in reticular nucleus (Landisman et al., 2002). In turn, they send their GABAergic inhibitory projections back to ventrobasal nucleus and POm (Scheibel and Scheibel, 1966; Pinault et al., 1995; Lam and Sherman, 2007). These inhibitory back-projections can account for the inter-barreloid inhibition in VPM (Desilets-Roy et al., 2002; Lavallée and Deschênes, 2004). While the topographic organization of the reticular neurons that project to VPM is somatotopic, no somatotopic map was found in the reticular neurons projecting to POm (Pinault et al., 1995).

In addition to thalamic reticular nucleus, a group of thalamic nuclei—termed extra-reticular inhibitory system—innervate POm with prominent GABAergic inhibitory projections (Bokor et al., 2005; Lavallée et al., 2005). The extra-reticular inhibitory system includes the anterior pretectal nucleus (APT) (Bokor et al., 2005), zona incerta (Barthó et al., 2002; Trageser and Keller, 2004; Lavallée et al., 2005) and pars reticulate division of substantia nigra (Buzsaki, 2009). Zona incerta (ZI) and APT are reciprocally connected, both project to PO and brainstem motor centers and receive layer V cortical inputs (Terenzi et al., 1995; May et al., 1997). ZI receives direct whisker input from both PrV and SpVi (Kolmac et al., 1998; Simpson et al., 2008) in addition to input from SI (Mitrofanis and Mikuletic, 1999; Barthó et al., 2007). Neurons in the dorsal and ventral divisions of ZI exhibit multi-whisker receptive fields (Nicolelis et al., 1992) with partial somatotopy in dorsal division and a complete somatotopic organization in ventral division (Nicolelis et al., 1992; Shaw and Mitrofanis, 2002). The ventral division of the zona incerta (ZIv) receives the main input from SpVi (Kolmac et al., 1998) and serves as a relay by feed-forward GABAergic inhibition of thalamocortical neurons in higher order thalamic nuclei including the paralemniscal pathway and POm for whisker-related motor activity (Trageser and Keller, 2004; Lavallée et al., 2005). The activation of vibrissal motor cortex suppresses vibrissal responses in ZIv (Urbain and Deschênes, 2007), providing a dis-inhibition mechanism for sensory gating in higher order thalamic nuclei during whisker-related motor activity and active touch. For a thorough review refer to Mitrofanis (2005).

### 2.6. Barrel Field Cortex

**Figure 2:**
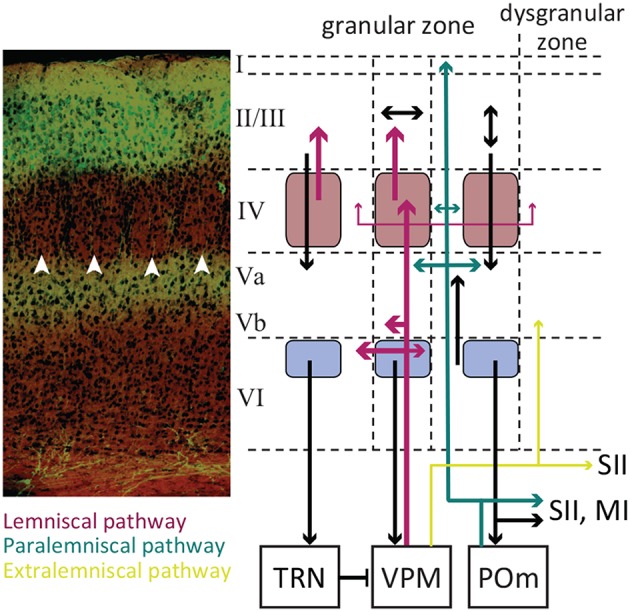
The laminar organization of SI. A coronal section of the somatosensory cortex with cresyl violet Nissl Staining (left panel). The white arrowheads indicate barrels in layer IV. Excitatory neurons in layer II/III are GFP labeled with their terminals in Layer Va. The laminar pathway containing glutamatergic excitatory projections from VPM to layer IV and sparsely to layers Vb and VI (labeled red). The paralaminar pathway containing the projections from POm to layer Va and I (labeled cyan). The pink boxes represent the barrels, and the light purple boxes represent infrabarrels. Adopted from Petersen (2007) and modified. Immunohistology and confocal microscopy image by Ehsan Kheradpezouh and Mehdi Adibi.

The cortical vibrissae representation in rodents is formally referred to as the posterior-medial barrel sub-field (PMBSF) and occupies about 20% of the somatosensory cortex (Zucker and Welker, 1969; Welker, 1971). The cortex is organized in 6 layers (Figure 2). In rodents, Layer IV of the vibrissae region of primary somatosensory cortex—referred to as the granular zone—contains anatomically distinguishable clusters of neurons called “barrels” (Woolsey and van der Loos, 1970). Each elliptically shaped barrel is approximately 0.3–0.5 mm in maximal diameter (Hodge et al., 1997) and contains an average of 2500 neurons (Woolsey and van der Loos, 1970; Lee and Woolsey, 1975; Jones and Diamond, 1995). Barrels are somatotopically arranged in an identical order as the whiskers on the snout, with the most dorsal posterior whiskers being represented by the most lateral posterior barrels (Woolsey and van der Loos, 1970). Neurons within each barrel produce their strongest and fastest response to the stimulation of the anatomically-associated whisker, also known as the “principal” whisker (Welker, 1971). There is a precise one-to-one connection between thalamic barreloids and cortical barrels, with no evidence of a multi-barrel innervation by thalamocortical axons (Bernardo and Woolsey, 1987; Chmielowska et al., 1989; Agmon et al., 1995; Land et al., 1995). In rats, there are sparse-celled regions between barrels called septa (Woolsey and van der Loos, 1970; Welker and Woolsey, 1974). Inter-barrel septa together with regions surrounding the barrel field form the dysgranular zone.

There are two main types of neurons in layer IV barrels: spiny stellate and star-pyramidal excitatory neurons, and GABAergic interneurons. Both excitatory and inhibitory neurons receive direct inputs from VPM. Neurons in layer IV heavily project into supragranular layer II/III within the same cortical column (along the barrel). Septal neurons project above septum to layer II/III and to some extent coarsely to surrounding barrels, secondary somatosensory cortex (SII) and primary motor cortex (Feldmeyer et al., 1999; Kim and Ebner, 1999; Petersen and Diamond, 2000; Chakrabarti and Alloway, 2006). Some layer IV barrel axons innervate into the adjacent barrels as well (Kim and Ebner, 1999; Petersen and Diamond, 2000; Brecht and Sakmann, 2002). The targets of layer II/III neurons include the adjacent barrel layer II/III, layer V, primary and secondary motor cortices, secondary somatosensory cortex, dysgranular zone, perirhinal temporal association cortex, dorsolateral striatum and the contralateral SI (Koralek et al., 1990; Hayama and Ogawa, 1997; Kim and Ebner, 1999; Yamashita et al., 2018). The laminar organization of neurons along a barrel form functional barrel columns across cortical layers which mainly represent the barrel's principal whisker.

Thalamic afferents innervate layer Vb and VI neurons concurrently to layer IV neurons (Constantinople and Bruno, 2013). Their synapses with layer V pyramidal neurons reliably elicit action potentials (Constantinople and Bruno, 2013). Axons of the layer V pyramidal neurons ramify extensively within this layer with ascending collaterals targeting the supragranular layers and descending collaterals projecting to infragranular layer VI (Thomson and Bannister, 2003; Lübke and Feldmeyer, 2010; Feldmeyer, 2012; Ramaswamy and Markram, 2015). Layer Va is predominantly populated by slender-tufted pyramidal neurons characterized by their slender apical dendrites, while layer Vb is predominantly populated by thick-tufted pyramidal neurons characterized by pyramidal-like somas and thick apical dendrites and the untufted pyramidal cells (Ramaswamy and Markram, 2015). The pyramidal neurons in layer Va (both slender and thick-tufted) may function as integrators of lemniscal and paralemiscal thalamic pathways through monosynaptic connections with layer IV spiny stellate neurons (Feldmeyer et al., 2005). The layer Vb thick-tufted pyramidal neurons mainly project to anterior midbrain and thalamic nuclei, including the posterior thalamus, ZI and APT. These projections maintain the somatotopic organization beyond the cortex (Sumser et al., 2017). For a detailed recent review of the neuroanatomy and physiology of the layer V refer to Ramaswamy and Markram (2015).

Layer VI is the main source of corticothalamic feedback projections (Bourassa et al., 1995; Feldmeyer, 2012). Corticothalamic neurons, in addition to projections to sensory thalamic nuclei, ramify both excitatory and inhibitory neurons in layer IV as well as pyramidal neurons in layer Va (Feldmeyer, 2012; Harris and Mrsic-Flogel, 2013; Kim et al., 2014). Paired whole-cell recording (Lefort et al., 2009) and laser scanning photo-release of caged glutamate (Hooks et al., 2011) revealed layer VI inter-laminar input and output are weak. However, repetitive optogenetic excitation of layer VI corticothalamic neurons evokes action potentials in layer Va pyramidal neurons as well as fast-spiking interneurons in both layer IV and Va by activating facilitating synapses (Kim et al., 2014), while the overall effect on layer IV excitatory neurons is weak excitation or disynaptic inhibition (Kim et al., 2014). Layer VIa corticothalamic neurons form aggregated barrel-like structures (called infrabarrels) organized somatotopically align with the layer VI barrels (Crandall et al., 2017). Corticocortical neurons, on the other hand, predominantly populate between infrabarrels. By optogenetic stimulation of VPM and POm thalamic nuclei, Crandall et al. (2017) found VIa corticocortical neurons receive strong synaptic input from both VPM and POm, whereas corticothalamic neurons exhibit weaker responses to VPM input and little response to POm. The receptive field properties of neurons in the barrel field are different across layers. The receptive fields in general have an excitatory center and excitatory surround structure; cortical neurons respond vigorously to the corresponding principal whisker as well as to the adjacent/surrounding whiskers with a weaker and delayed response (Simons, 1978; Armstrong-James and Fox, 1987). However, septal neurons similar to their presynaptic POm neurons, respond to multiple whiskers without preference to a certain whisker as principal (Armstrong-James and Fox, 1987; Brecht and Sakmann, 2002). Consistent with their pattern of connectivity, layer II/III neurons, show a broader receptive field with a lower response magnitude (Ito, 1985; Armstrong-James and Fox, 1987; Armstrong-James et al., 1992). Synaptic integration in layer V neurons is more complex, as these neurons receive input from layers II/III (Reyes and Sakmann, 1999), IV (Feldmeyer et al., 2005; Schubert et al., 2006), from other pyramidal neurons in the infragranular layers (Markram et al., 1997; Schubert et al., 2001), as well as substantial direct thalamic input (Bureau et al., 2006). This leads to broad receptive fields and sometimes whisker non-specific response profiles (Sachdev et al., 2001). For a more detailed review on SI laminar organization refer to Ahissar and Staiger (2010). Also, for a review on the functional organization of barrel cortex refer to Petersen (2007).

Across all cortical laminae, increasing the velocity/acceleration of stimuli applied to the principal whisker increased the amplitude of excitatory post synaptic potentials (EPSPs) and decreased their latency to peak (Wilent and Contreras, 2004). The changes in the EPSP were accompanied by a transient increase in the spiking activity of cortical neurons (Simons, 1978; Ito, 1985; Pinto et al., 2000; Arabzadeh et al., 2003; Wilent and Contreras, 2004; Adibi and Arabzadeh, 2011), typically followed by a rapid decline (within 10–20 ms of the response onset) to a lower level of tonic spiking rate. The synaptic response of supragranular (layer II/III) and infragranular (layer V and VI) neurons was on average delayed with respect to that of the granular (layer IV) neurons (Brecht and Sakmann, 2002; Brecht et al., 2003; Manns et al., 2004; Wilent and Contreras, 2004, but see Constantinople and Bruno, 2013). The peak of the spiking response of Layer IV neurons was followed by infragranular neurons' response peak and then by the response peak of layer II/III neurons (Wilent and Contreras, 2004). Layer IV neurons exhibit a short integration window of a few milliseconds compared to other layers. These findings suggest that layer IV neurons function as coincidence detectors, whereas supra- and infragranular circuits function as input integrators (Wilent and Contreras, 2004; Brecht, 2007). Layer V neurons are proposed to integrate lemniscal and paralemniscal inputs in addition to inputs from most or all cortical layers (Brecht, 2007). Layer IV, III and II, on the contrary, might operate as functionally segregated circuits contributing to separate lemniscal and paralemniscal processing streams (Brecht, 2007).

**Figure 3:**
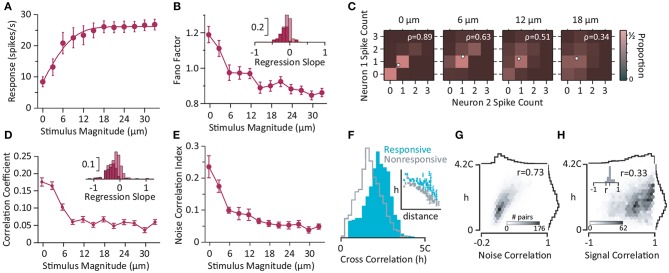
Neural activity in somatosensory cortex. **(A)** The population activity increases with the magnitude of whisker deflection stimulation (single-cycle sine-wave at 80 Hz). Error bars represent the standard error of means across populations with more than five simultaneously recorded units (*n* = 8). **(B)** Trial-to-trial variations in neuronal response (in terms of Fano factor) as a function of stimulus intensity for single neurons (*n* = 64). The inset depicts the histogram of the linear regression slope of the Fano factor with respect to the z-scored neuronal activity for individual neurons. The dark bars correspond to recordings with a significant linear regression (*p*<0.05). **(C)** Color indicates the proportion of joint spike counts for a pair of simultaneously recorded neurons. White circles indicate mean spike counts for each stimulus. The Pearson's correlation coefficient of the spike counts is indicated by ρ for each panel. **(D)** The mean Pearson's correlation coefficient across all possible pairs of neurons (*n* = 245) as a function of stimulus intensity. Error bars indicate standard error of means. The inset depicts the histogram of regression slopes of noise correlation against average firing rate for pairs of neurons. Dark bars indicate the cases with significant linear regression (*p*<0.05). **(E)** The noise correlation index (Adibi et al., 2013b) as a function of stimulus magnitude averaged across populations containing at least five simultaneously recorded neurons (data from **A**). Error bars are standard error of mean across populations (*n* = 8). Most of the neurons exhibit a negative slope indicating Fano factor **(B)** and noise correlations decrease with firing rate. **(F)** The strength of correlation, denoted by h: the peak of the cross correlation of a pair of electrodes relative to the chance level (denoted by C). Electrodes were divided into two groups of “Responsive” and “Nonresponsive” based on the median of the mutual information between neuronal responses and whisker stimulation. The distribution of h values for Responsive pairs (where both electrodes were from the Responsive group; cyan) and Nonresponsive pairs (where both electrodes in a pair were from the Nonresponsive group; gray). The inset depicts the average and standard error of means of strength of correlation, h, across electrode pairs as a function of their distance for each category. **(G)** The histogram shows the joint distribution of h values and noise correlations. r represents the correlation coefficient. **(H)** Same as **(G)**, but for signal correlation. Inset depicts the histogram of r value calculated for groups of electrode pairs with identical distance. The distribution of r values is positive with a mean of 0.3 indicating that the positive correlation between h and signal correlation is independent of the distance between electrodes and is present across all distances. **(A–E)** are based on Adibi et al. (2013b), and **(F–H)** are from Sabri et al. (2016).

The sequence of cortical activation across layers is consistent with interlaminar interacortical local field potential recordings and current source analysis which exhibit early current sinks in layer IV followed by activation of layers II/III and V (Di et al., 1990; Agmon and Connors, 1991; Kenan-Vaknin and Teyler, 1994). Multi-electrode array electrophysiology from SI neurons revealed whisker deflection stimulation quenches trial-by-trial variability (Adibi et al., 2013b); the Fano factor, defined as the ratio of the variance of neuronal responses to their average, decreased as the stimulus intensity (and hence the population activity) increased (Figures 3A,B). This decrease is consistent with previous findings in areas V4 (Cohen and Newsome, 2009) and MT (Uka and DeAngelis, 2003; Osborne et al., 2004), premotor cortex (Churchland et al., 2006), and superior temporal sulcus (Oram, 2011) of monkeys (for a detailed review see Churchland et al., 2010). Stimulation quenches the correlation in trial-to-trial variability between neurons (noise correlation) (Figures 3C,D). Noise correlation is usually characterized in terms of the correlation coefficient of the spike counts for pairs of neurons. Using principal component analysis of neuronal responses, Adibi et al. (2013b) extended this measure to neuronal populations of larger than 2 neurons (see Figure 3E). The functional connectivity map constructed based on the strength of pairwise correlations of ongoing spontaneous activity of urethane-anesthetized rats recorded using 10 × 10 array of electrodes predicted the anatomical arrangement of electrodes on the sensory cortex (Sabri et al., 2016). Neurons with stronger correlations to the population during episodes of spontaneous activity, carried higher information about the sensory stimuli in their evoked response (Figure 3F). It is, however, not clear whether this higher level of correlations is due to common input from thalamus or originates from the cortical circuitry. Moreover, the correlation profile of electrode pairs during spontaneous activity predicted both signal and noise correlations (Adibi et al., 2014) during sensory stimulation (Figures 3G,H).

It has been demonstrated that barrel cortex neurons in anesthetized rats robustly encode the velocity of whisker motion (Simons, 1978; Pinto et al., 2000; Arabzadeh et al., 2003, 2004; Estebanez et al., 2012). The whisker motion features that these neurons encode form a common low-dimensional feature subspace of whisker motion, comprising linear combination of whisker velocity and position, and to a lesser extent whisker acceleration (Maravall et al., 2007; Estebanez et al., 2012). Estebanez et al. (2012) recently demonstrated that the feature encoding properties of cortical neurons differ depending on the level of spatial correlation in multi-whisker sensory stimuli. In addition to velocity, cortical neurons in the whisker-related area of SI exhibit directional selectivity (Simons, 1978; Simons and Carvell, 1989; Bruno and Simons, 2002; Wilent and Contreras, 2005; Puccini et al., 2006; Kremer et al., 2011).

The feedback projections from infragranular layers to the vibrissae-related thalamic sensory nuclei consist of three main routes:

1. Neurons in the upper part of layer VI of a barrel exclusively project to the corresponding barreloid in VPM (Bourassa et al., 1995; Land et al., 1995) forming a reciprocal barreloid-barrel connection.
2. Neurons in the lower part of layer VI project to POm and also a major proportion of these axons make collaterals in VPM to form rostro-caudal rod-like bands representing an arc of vibrissae (Hoogland et al., 1987; Bourassa et al., 1995).
3. The corticothalamic projections of layer V cells exclusively terminate in POm (Bourassa et al., 1995).

The axons originated from layer VI along the inter-barrel septa exclusively target POm (Bourassa et al., 1995). The Layer VI corticothalamic axons, but not those of layer V give off collaterals in the reticular nucleus while traversing it (Bourassa et al., 1995; Deschênes et al., 1998).

The primary somatosensory cortex projects to the secondary somatosensory cortex, the primary motor cortex (MI), thalamus sensory nuclei, superior caliculus and dorsolateral neostriatum (White and DeAmicis, 1977; Carvell and Simons, 1986, 1987; Welker et al., 1988; Deschênes et al., 1998; Alloway et al., 2006; Chakrabarti and Alloway, 2006; Hattox and Nelson, 2007; Larsen et al., 2007). Also, the barrel cortices on two hemispheres are linked by a callosal connection (White and Czeiger, 1991). In turn, primary somatosensory cortex receives inputs from the secondary somatosensory cortex and motor cortex (Carvell and Simons, 1987; Kim and Ebner, 1999). Unlike in primates (Hsiao et al., 1993; Jiang et al., 1997; Iwamura, 1998; Mima et al., 1998; Karhu and Tesche, 1999; Salinas et al., 2000; Romo et al., 2002), little is known about the functional properties of the secondary somatosensory cortex in rodents, and this knowledge is limited to anesthetized preparations (Carvell and Simons, 1986; Kwegyir-Afful and Keller, 2004).

### 2.7. Parallel Ascending Subcortical Routes for Whisking and Touch Signals to Cortex

The whisker information from trigeminal complex is channeled to cortex through three parallel pathways (Pierret et al., 2000; Yu et al., 2006, also see Figures 1, 2):

1. The lemniscal pathway is the major pathway through which the touch signal is channeled to cortex. This pathway includes ipsilateral PrV barrelettes to contralateral VPMdm barreloids to cortical barrel columns layer IV and sparsely to Layer VI in SI. The lemniscal pathway conveys a combination of touch and whisking signals and is speculated to represent the “what” pathway (analogous to the ventral stream in the visual system).
2. The paralemniscal pathway channels the sensory information from rostral part of alaminar spinal trigeminal nucleus (nucleus interpolaris or SpVi) into the thalamic posterior medial nucleus (POm), and then to the following cortical areas: layer I and Va of SI, the septal regions, SII, MI and superior colliculus. The paralemniscal pathway primarily conveys whisking signals, which can be employed to form sensory-motor coordination and positional reference signals during exploration/whisking (Ahissar et al., 2000; Kleinfeld et al., 2006). Hence the paralemniscal pathway is speculated to represent the “where” system in somatosensation in rodents (analogous to the dorsal stream in the visual system).
3. The extralemniscal pathway conveys touch information from SpVc and caudal division of SpVi to VPMvl thalamus and then to SII and the septal regions of SI cortex.

The lemniscal and paralemniscal pathways interact; the lemniscal pathway has been shown to suppress the paralemniscal pathway through cortically-activated rapid GABAergic inhibitory projections of zona incerta to POm (Lin et al., 1990; Nicolelis et al., 1992; Power et al., 1999).

## 3. Physiology and Function

### 3.1. Modes of Whisker-Mediated Sensation

As in vision where controlled eye movements—saccades—enhance the efficacy of the visual system to browse the environment and extract relevant visual information, rodents sweep their mystacial vibrissae to scan the environment and collect behaviorally-relevant information. A body of literature referred to this purposively information-seeking manipulation of sensory apparatus as “active sensing” (Gibson, 1962; Aloimonos et al., 1988; Aloimonos, 1990; Szwed et al., 2003, 2006; Mitchinson et al., 2007; Grant et al., 2009; Sullivan et al., 2012). In the realm of engineering, however, “active sensing” against “passive sensing” means emitting energy (e.g., in electromagnetic form as in radar or in mechanical form as in sonar) and sensing the reflections of the emitted signal to obtain information about the medium/environment. To avoid this ambiguity, here, I follow the terminology as in Diamond and Arabzadeh (2013) which categorize the whisker-mediated perception in rodents into two modes: “generative” and “receptive.”

Whisking is the self-generated exploratory whisker motion through which rodents sense their surrounding environment in the “generative mode.” This generative mode of whisking is used in the perception of surface textures, identification of objects and shapes, estimation of distances and localization of objects. As a whisker comes in contact with an object or palpates the object, its instantaneous motion changes following every contact and release from the surface with high acceleration and high velocity—stick-slip events. The sequence of these stick-slip events along with the self-generated component of the whisker motion uniquely reconstructs the kinetics of surface and determines the texture of a surface, or the shape or location of an object. A body of research has focused on quantification of behavioral capacities and characterization of whisker motion and its consecutive neuronal activity in the generative mode. These include a variety of behavioral tasks or simulated conditions such as texture discrimination (Carvell and Simons, 1990; Guić-Robles et al., 1992; Prigg et al., 2002; Arabzadeh et al., 2005; von Heimendahl et al., 2007; Diamond et al., 2008; Itskov et al., 2011; Morita et al., 2011; Zuo et al., 2011), identification of shape and size of objects (Brecht et al., 1997; Harvey et al., 2001; Polley et al., 2005), distance, gap and aperture width detection (Hutson and Masterton, 1986; Guic-Robles et al., 1989; Harris et al., 1999; Jenkinson and Glickstein, 2000), object localization (Knutsen et al., 2006; Mehta et al., 2007; Ahissar and Knutsen, 2008; Knutsen and Ahissar, 2009; O'Connor et al., 2010) and natural exploratory whisking (Fee et al., 1997; Kleinfeld et al., 2002, 2006; O'Connor et al., 2002; Berg and Kleinfeld, 2003; Szwed et al., 2003; Ganguly and Kleinfeld, 2004; Knutsen et al., 2005). For other paradigms, such as width discrimination described in (Krupa et al., 2001) whisking may not be essential. However, I categorized such behavioral tasks in the generative mode as they require controlled head positioning and movements.

As in vision where fixating the gaze on a focal target provides more accurate visual information, in receptive mode, rats can immobilize their vibrissae to achieve efficient vibro-tactile signal collection from a mobile object. In vision, saccades during a fine visual task such as counting degrade the performance. Similarly, there is behavioral evidence that self-generated whisker motion reduces the rodent's performance when detecting vibrations (Ollerenshaw et al., 2012). This aspect of whisker-mediated sensation is less investigated in the literature (Hutson and Masterton, 1986) and research has been mainly limited to head-fixed rodents performing a go/no-go licking task (Stüttgen and Schwarz, 2008, 2010; Gerdjikov et al., 2010; Schwarz et al., 2010).

Recent studies revealed that the response dynamics of cortical neurons changes with the mode of sensation and the behavioral state (Fanselow and Nicolelis, 1999; Castro-Alamancos, 2004; Crochet and Petersen, 2006; Ferezou et al., 2006, 2007). The response of cortical neurons to whisker stimuli was suppressed in the generative mode compared to the receptive mode or quiescent state (Castro-Alamancos, 2004; Crochet and Petersen, 2006; Ferezou et al., 2006, 2007; Crochet et al., 2011). Likewise, neurons in rat auditory cortex show sensory-evoked response suppression during active behavioral states (Otazu et al., 2009). Additionally, fluctuations in local field and membrane potentials of layer II/III cortical neurons exhibit prominent slow synchrony during receptive mode (Crochet and Petersen, 2006; Poulet and Petersen, 2008; Gentet et al., 2010, 2012). In the generative mode during free whisking, however, membrane potential fluctuations were suppressed and desynchronized across nearby neurons. This cortical state of desynchrony was accompanied by an increase in the spiking activity of thalamocortical neurons (Poulet et al., 2012). Cutting the sensory peripheral afferents innervating whisker follicles did not affect the generative mode response suppression and desynchrony, indicating that its origin is not peripheral (Poulet et al., 2012). Pharmacological inactivation of thalamocortical neurons, however, halted the generative-mode desynchronization. Consistently, optogenetic stimulation of thalamocortical neurons induced similar desynchronized cortical state (Poulet et al., 2012). For further details refer to the review article by Petersen and Crochet (2013).

### 3.2. Behavioral Approaches to Systems Neuroscience: Linking Circuitry and Function

How does neuronal activity give rise to sensation and ultimately perception? To what extent does the neuronal readout match the perception of whisker vibration? In order to draw a causal link between neuronal activity and sensorimotor, perceptual, and cognitive functions, it is crucial to develop appropriate behavioral methods and combine them with requisite methods of observation and perturbation of neuronal activity. The behavioral approaches in rodent model system are either based on native forms of natural behavior such as whisking, hence require minimum training—for instance, free navigation or exploration, whisking and aperture or gap crossing (Harris et al., 1999; Jenkinson and Glickstein, 2000; Crochet and Petersen, 2006; Celikel and Sakmann, 2007; Sofroniew et al., 2014; Kandler et al., 2018)—or paradigms embedded in an artificial task and require extensive training of the animal to interact with the environment and express specific behaviors in response to events and stimuli—in this context, neutral tactile stimuli such as textures, vibrations or object contacts. The body of literature mainly divides into two forms of behavioral tasks: (i) go/no-go or lick/no-lick, and (ii) two- or multiple-alternative-choice tasks.

Go/no-go (or lick/no-lick) tasks are often used in the head-fixed preparation predominantly in mice and sometimes in rats (Topchiy et al., 2009; Schwarz et al., 2010; Guo et al., 2014a; Fernández et al., 2018a; Helmchen et al., 2018). It provides the mechanical stability and a fixed head position ideal for precise whisker stimulation, whisker motion tracking, eye/pupil and gesture tracking, as well as electrophysiology (for instance, intracellular recording) and imaging from cortex (two-photon calcium imaging or voltage-sensitive dye imaging). To prevent learning about timing of the reward as a confounding cue, and to minimize impulsive or anticipatory responses based on the periodicity of the sensory events and reward, go/no-go tasks usually do not have a discrete trial structure, or the initiation of a trial is at random time instances with variable delays. The proportion of the trials followed by no-go should be precisely balanced in order to minimize excessive reinforcement of spontaneous incorrect go choices (false alarms) and to avoid formation of a bias toward go or no-go choices. Other limitations of the go/no-go tasks in head-fixed preparation include no re-enforcement (reward) for correct no-go choices, suppressed vestibular signals which may play a crucial role for coordination of whisking behavior and body movements, and relying on licking behavior with highly reflexive components (Keehn and Arnold, 1960; Schaeffer and Premack, 1961; Hulse and Suter, 1968) as a representation of a cognitive goal-directed behavior. Using conditioned level-press responses, Mehta et al. (2007) found that rats with only a single whisker combine touch and whisker movement to distinguish the location of objects at different angular positions along the sweep of whisker. The other limitation of go/no-go head-fixed tasks is the lack of control over motivational factors (e.g., satiation) affecting the likelihood of go choices. The motivation can be controlled by employing a self-initiation mechanism for trials. Go/no-go paradigm is commonly used to quantify the behavioral performances for detection of a stimulus or the detection of change (Stüttgen and Schwarz, 2008; Ollerenshaw et al., 2012; Bari et al., 2013) and discrimination of two sets of stimuli, one associated with go (and hence reward), and one associated with no-go (Mehta et al., 2007; Gerdjikov et al., 2010; O'Connor et al., 2010; Chen et al., 2013). Lee et al. (2016) applied a visuo-tactile detection go/no-go task in freely moving rats with the minimum level of temporal uncertainty; upon the initiation of a trial by nose-poke into a port, the sensory cue (whisker deflection or visual flicker) appeared after a delay of either 300 or 800 ms each of which with equal likelihood. After stimulus onset, the rat had a 500 ms window of opportunity to elicit the go choice and collect the reward. For a hypothetically “logical” rat, the optimal strategy is to detect the sensory stimulus only at the time instance associated to the short delay (300 ms). Upon no detection at 300 ms, the hypothetical rat makes an anticipatory non-sensory go choice at 800 ms, as the hazard rate for stimulus presentation (and hence reward delivery) at 800 ms equals 1 (i.e., absolute certainty). This non-sensory anticipatory strategy explains the faster response time to 800-ms stimulation compared to 300-ms stimulation observed in (Lee et al., 2016). Additionally, this strategy predicts a higher proportion of misses for short delay stimulation and higher hit rate for the long delay stimulation (see also Lee et al., 2019). Extracellular array recording from vSI neurons during this task revealed enhanced cortical activity to whisker stimulation with higher expectancy (likelihood compared to visual stimulus) (Lee et al., 2016, 2019). This supports a plausible multiplicative gain modulation of evoked responses or alternatively an additive modulation of baseline activity. This response enhancement may be induced by expectation or attentional factors, motor preparation or sensory events related to motor output (as the task lacks a delay after stimulus presentation to withhold the go choice and to separate stimulus presentation from choice), decision processes and motor output. This is a common drawback in go/no-go, and in particular, lick/no-lick paradigms. In contrast to go/no-go tasks in which it is difficult to distinguish a lack of motivation or lapses of attention from false rejections or correct rejections, two-alternative-choice tasks provide a clear distinction of correct, incorrect, and missed trials.

**Figure 4:**
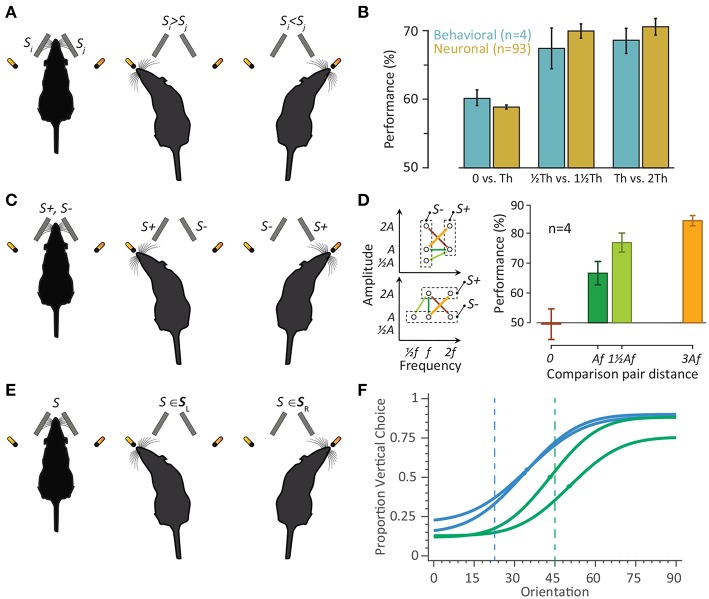
The two-alternative-choice behavioral tasks in rodents. **(A)** Schematic representation of the comparative discrimination paradigm. On every trial, two vibrations *S*_*i*_ and *S*_*j*_ were presented. **(B)** Four rats were trained in the detection/discrimination task to identify the vibration with the higher amplitude. The neuronal performance is the average performance (based on the area under ROC) across single-units (*n* = 35) and multiunit clusters (*n* = 58) from Adibi and Arabzadeh (2011). For each neuron, the stimulus intensity whose detection performance was closest to 60% was chosen as detection threshold (*Th*). The stimuli corresponding to $\frac{1}{2}$−, $1\frac{1}{2}$−, and 2-fold *Th* were then selected for estimating the discrimination performances. The same threshold of 60% defined as detection threshold for rats. The rats performed the comparison task between 0−*Th*, $\frac{1}{2}-1\frac{1}{2}$ and *Th* − 2*Th*. Error bars indicate standard error of means across rats or neurons. **(C)** Schematic representation of the categorical discrimination paradigm. Stimuli were defined as either S+ or S−. In each trial, one of the two vibrations was S+ and the other was S−. Having identified the S+ vibration, the rodent expressed its choice by turning toward the corresponding drinking spout. **(D)** (Left) Stimulus space. Each circle represents the frequency–amplitude combination of one stimulus. Two groups of rats were trained in the task. For one group (top-left), two frequencies (*f* = 80 Hz and 2*f* = 160 Hz) and three amplitudes ($\frac{1}{2}A$ = 8 μm, *A* = 16μm, and 2*A* = 32 μm) were used to generate five vibrations, and for second group (bottom-left) three frequencies ($\frac{1}{2}f$ = 40 Hz, *f* = 80 Hz and 2*f* = 160 Hz) and two amplitudes (*A* = 16 μm and 2*A* = 32 μm) were used to generate five vibrations. Stimuli that were presented together and had to be discriminated (paired stimuli) are connected by lines. The right panel shows the proportion of correct trials (performance) for the corresponding four stimulus-pairs averaged across rats. Error bars are s.e.m. across rats. Re-plotted from (Adibi et al., 2012). **(E)** The schematic representation of the categorization paradigm. The stimuli are divided into two categories of *S*_*L*_ and *S*_*R*_, corresponding to left and right choices, respectively. A stimulus *S* was presented on every trial. The rat identifies the category which stimulus *S* belongs to. **(F)** Rats were trained to categorize the orientation of a 9.8 cm-diameter disc with alternating ridges and grooves by licking at one of the two reward spouts. Psychometric functions correspond to two rats trained to categorize orientations 0–45° as horizontal, and 45–90° as vertical (green), and another two rats trained to categorize orientations 0–22.5° as horizontal, and 22.5–90° as vertical (blue). The curves correspond to a Gaussian cumulative function fitted to data. The dots on each curve represent the perceptual decision boundary of each rat. The blue and green vertical dashed lines represent the categorization boundaries of 22.5° and 45°, respectively.

Two- or multiple-alternative-choice tasks can be divided into two main categories: sensory discrimination/comparison and categorization tasks (Figure 4). In sensory discrimination tasks, every trial includes presentation of two stimuli. Discrimination/comparison tasks take two forms depending on the association of the two choices with the stimuli. In the “comparative” discrimination (Figure 4A), the task is to compare an attribute of the two stimuli against each other [e.g., roughness of textures (Carvell and Simons, 1990), frequency (Adibi et al., 2012; Mayrhofer et al., 2012), magnitude (Adibi and Arabzadeh, 2011; Adibi et al., 2012; Fassihi et al., 2014, 2017), or duration (Fassihi et al., 2017) of two vibrations]. Each outcome of the comparison is associated with one of the two reward ports. The two stimuli may present simultaneously at two distinct positions (e.g., two whiskers, or two sides of snout Carvell and Simons, 1990; Adibi and Arabzadeh, 2011; Adibi et al., 2012) or at one position but at distinct time instances (Fassihi et al., 2014, 2017). In the “categorical” discrimination (Figure 4C), the stimuli are divided into two categories of rewarded/target (S+) vs. unrewarded/distractor (S−). Each trial comprises presentation of one stimulus from each of the two categories. The task is to select the choice associated to the position of the target/rewarded stimulus (Morita et al., 2011; Adibi et al., 2012; Mayrhofer et al., 2012; Musall et al., 2014).

In the categorization tasks, the stimuli are divided into two categories, each of which associated with one of the two choices (Figure 4E). On every trial, one stimulus is presented, and the task is to identify the category to which the stimulus belongs. Categorization tasks can be considered as a discrimination/comparison task against a reference or boundary dividing the physical feature space of the stimulus into two categories. Alternatively, it can be considered as a mapping of individual stimuli with one of the two choices. Rodents can perform whisker-mediated tactile categorization tasks on sensory attributes such as textures (von Heimendahl et al., 2007; Zuo et al., 2011; Grion et al., 2016; Zuo and Diamond, 2019b), whisker deflection amplitude pattern (McGuire et al., 2016), aperture width (Krupa et al., 2001), location (Guo et al., 2014b; Li et al., 2015; Helmchen et al., 2018) and orientation (our recent data in Figure 4F, also see Nikbakht et al., 2018) of objects.

Discrimination and detection behavioral studies quantify the psychometric response function (the likelihood of the choices as a function of stimulus attribute) which along with the acquisition of neuronal activity allows linking the behavioral function to the neuronal activity. Comparison of the neuronal and psychophysical performances started in the late 1960s in the classic electrophysiological experiments in cat retina (Barlow and Levick, 1969; Barlow et al., 1971) and in the somatosensory cortex (Talbot et al., 1968; Mountcastle et al., 1972). Thereafter, more studies have combined psychophysical and neurophysiological experiments in order to relate neuronal responses to perception (Romo et al., 1998, 2000; Hernández et al., 2000; Salinas et al., 2000; Ress and Heeger, 2003; Luna et al., 2005; de Lafuente and Romo, 2006; Stüttgen and Schwarz, 2008) and decision making (Newsome et al., 1989; Shadlen et al., 1996; Romo et al., 2004; Hanks et al., 2006; Kiani et al., 2008). Instead of the traditional comparison of behavioral and neuronal thresholds or sensitivities, Adibi and Arabzadeh (2011) compared the non-linearity of the behavioral and neuronal response profiles to the amplitude of vibration. In a series of vibration detection and amplitude discrimination tasks, Adibi and Arabzadeh (2011) first quantified the detection threshold of both cortical neurons and rats (denoted by *Th*, Figure 4B). For near-threshold stimuli with identical amplitude difference, both the neuronal and behavioral discrimination performances surpassed the detection performances (Figure 4B). This is consistent with the accelerating nonlinearity of neurometric and psychometric functions at low stimulus intensities. The results revealed the nonlinearity in the neuronal response function predicts behavioral detection and discrimination performances. This study presents the first observation of the “pedestal effect”—frequently reported in human psychophysics—in animal literature. Using the same behavioral detection task, McDonald et al. (2014) showed rats' behavior indicated a dynamic stimulus sampling whereby stimulus sampling was continued until the stimulus was correctly identified or the rat experienced a false alarm. This is consistent with the recent evidence from texture identification task (Zuo and Diamond, 2019a,b) suggesting similar to primates, rats' choices are governed by bounded integration of primacy-weighted touch-by-touch evidence.

Previous electrophysiology studies identified the physical features of whisker motion that are encoded in the activity of cortical neurons to be the product of elemental features of whisker motion, its frequency (*f*) and amplitude (*A*) (Simons, 1978; Ito, 1985; Pinto et al., 2000; Arabzadeh et al., 2003, 2004). Consistently, behavioral studies revealed rats are unable to discriminate these elemental features independently of their product (Adibi et al., 2012); two groups of rats were trained to discriminate either based on the frequency or based on the amplitude of the vibrations delivered to both whisker pads. The stimulus pairs with identical *Af* product (marked in red, Figure 4D) were not discriminable, while the other stimulus pair with the same feature difference in the physical space (marked with orange, Figure 4D) were highly discriminable. In both groups, rats' performance in discriminating two stimuli is accounted for by the difference in *Af* but not by differences in either elemental feature (*A* and *f*) alone. This is consistent with the electrophysiological findings that neurons reduced the dimensionality of the stimulus from two features (*A*, *f*) to a single feature: the product *Af* (Arabzadeh et al., 2003, 2004). *Af* defines a real physical property: the speed of whisker motion averaged over cycles.

The bridge linking neuronal activity to perception is the readout mechanism of sensory neurons. The interlaced synaptic architecture of neural networks provides strong evidence for decoding by downstream neuronal structures based on “populations” of neurons rather than individual single neurons. Such a synaptic organization together with physiological properties of dendritic processes by which neurons receive information simulates an integration model in which the activity of neurons in the relevant population is summed with different weights. This provides a simple framework to investigate how a biologically plausible ideal observer of neuronal responses, a linear “decoder,” extracts information about the stimuli. Linear decoders are simple in their structure and compatible with the architecture of the brain. With optimizing the weights, it provides an upper limit to the amount of information extractable from neuronal responses. There are two limiting factors affecting the reliability of the neuronal code for sensory stimuli: the response variability of individual neurons to a given stimulus, and co-variability (noise correlation) across the neurons. In our previous studies, we characterized the neuronal response statistics in terms of neuronal variability (Fano factor) and co-variability (noise correlation) and parsed out the effect of each of these components on the coding as well as decoding efficiency of cortical populations (Adibi et al., 2013a,b, 2014). Adibi et al. (2014) further quantified the effect of noise correlations on the optimal linear decoder and characterize the cost of ignoring noise correlations during decoding.

### 3.3. Motion Detection and Spatial Invariancy in Whisker-Mediated Touch System

A majority of neurons across different layers of the rat barrel cortex exhibit multi-whisker receptive fields (Simons, 1978; Armstrong-James and Fox, 1987; Moore and Nelson, 1998; Ghazanfar and Nicolelis, 1999; Brecht and Sakmann, 2002; Brecht et al., 2003). The spatial extent of the receptive field of a cortical neuron depends on the intra-cortical connections between barrel columns (Armstrong-James et al., 1991). Anatomical studies revealed that intra-cortical inter-barrel connections are stronger between barrels within a row (Bernardo et al., 1990a,b; Hoeflinger et al., 1995), with directionally-biased fiber projections into the anterior barrel (Hoogland et al., 1987; Bernardo et al., 1990a). Additionally, intra-cortical projections from septal columns extend two to three barrels along the rows (Kim and Ebner, 1999). Consistently, the activity pattern of VPM and cortical neurons to single-whisker deflections is elongated along rows (Simons, 1978; Armstrong-James and Fox, 1987; Armstrong-James et al., 1992; Lee et al., 1994; Kleinfeld and Delaney, 1996). Electrophysiological studies also revealed that the multi-whisker interaction along rows and arcs is not symmetric. Suppressive two-whisker interactions have been reported to be more prominent during within-row stimulation than during within arc stimulation (Ego-Stengel et al., 2005), while within-arc multi-whisker stimulation yields more supra-linear response integration (Ghazanfar and Nicolelis, 1997; Ego-Stengel et al., 2005). However, multi-whisker interactions are highly dependent upon the temporal order and timing of the stimulation (Shimegi et al., 1999, 2000). Estebanez et al. (2012) demonstrated that the feature encoding properties of cortical neurons changes with the level of spatial correlation in multi-whisker sensory stimuli. In addition to its anatomical and functional importance, the rostro-caudal axis is behaviorally relevant. Through exploratory behavior, rats whisk (move their vibrissae) rostro-caudally, leading to a functional asymmetry between rows and arcs; as the whiskers palpate an object, whiskers within a row contact the object successively relative to their rostro-caudal position in the row, whereas whiskers within an arc usually contact the object nearly simultaneously. Thus, a potential function of within-arc facilitatory interactions might be to boost up the contact signal which is more likely to arise from whiskers within an arc. Alternatively, the spatiotemporal multi-whisker interactions could be an indication of cross-whisker motion detection (e.g., head relative to environment and vice versa) at the level of neurons in the rat primary somatosensory cortex or secondary somatosensory cortex (Jacob et al., 2008). Simple biologically-plausible models such as the Reichardt model (Hassenstein and Reichardt, 1956; Reichardt, 1961)—a correlation detector based on temporal delays—or energy models (Adelson and Bergen, 1985) provide plausible frameworks underlying movement detection in barrel cortex. Such motion detectors are more likely to be identified in SII or in the infra-granular layers of SI where neurons have broad multi-whisker receptive fields. In addition to information about the velocity of moving objects or the ego motion, such motion detectors can provide information about the location of objects with respect to head during whisking or head movements. A recent study (Curtis and Kleinfeld, 2009) showed that barrel neurons provide a representation of the position of contacted objects in a coordinate frame that is normalized to the trajectory of the motor output (i.e., phase of whisking). Contact was encoded independently of the angular whisker position and was shown to be invariant with respect to the amplitude and frequency of whisking. The representation of contact in a coordinate system that is dynamically normalized by the motor output provides the basis for encoding the spatiotemporal properties of an externally induced movement.

Le Cam et al. (2011) demonstrated that functional principal whisker—the whisker eliciting the strongest response with the shortest latency—differed based on the direction of whisker deflection along the rostro-caudal axis. The stimulus-induced changes in the spatial structure of the receptive field of the neurons was not limited to the principal whisker, and included stimulus-dependent changes in the size, response latency and receptive field center of mass. Although the neuronal mechanisms underlying these dynamic changes are not clear, they suggest invariancy of whisker position through whisking along the rostro-caudal axis; as the rat whisks, the position of the whiskers changes along the rostro-caudal axis with respect to the head leading to potential ambiguity about the position of an object in contact with the whisker. Such a dynamic shift in the receptive fields might help to adjust the position of contact with respect to the head instead of the whisker. This position invariant information can potentially give rise to whisker-mediated coordination, and contribute to spatio-topic representations in grid cells (Hafting et al., 2005) in the entorhinal cortex, head-direction cells in classic Papez circuit (Taube, 1998) and place cells (O'Keefe, 1976; O'keefe and Conway, 1978; O'Keefe and Nadel, 1978) in parahippocampal and hippocampal cortices.

### 3.4. Directional Selectivity in Whisker-Mediated Touch System

There are several lines of evidence that cortical neurons in the whisker area of SI exhibit directional selectivity (Simons, 1978; Simons and Carvell, 1989; Bruno and Simons, 2002; Wilent and Contreras, 2005; Puccini et al., 2006; Kremer et al., 2011; Kwon et al., 2018). Direction preference is also observed in the response of thalamic and trigeminal neurons (Shosaku, 1985; Lichtenstein et al., 1990; Hartings et al., 2000; Minnery et al., 2003; Timofeeva et al., 2003; Furuta et al., 2006; Bellavance et al., 2010). Although the directional selectivity in the periphery and brainstem originates in the uneven arborization of nerve terminals around the follicle (Lichtenstein et al., 1990), direction-dependent differences in the temporal profile of synaptic excitation and inhibition in barrels (Wilent and Contreras, 2005) and non-linear dendritic processes (Lavzin et al., 2012) also may contribute to the directional tuning in barrel cortex neurons. The directional selectivity decreases along the ascending whisker-to-barrel pathway. The functional and behavioral correlate of directional selectivity in the whisker-to-barrel system is not understood and it is not clear whether rats perceive the direction of vibro-tactile stimulus. However, several lines of research provide evidence against an angular selectivity readout such that leads to a sensation of direction. First, neurons with multi-whisker receptive fields in cortex and thalamus do not necessarily exhibit the same angular preference to different whiskers in their receptive field (Hemelt et al., 2010, but see Kida et al., 2005). Second, in the visual system, orientation selectivity arises from specific convergence of directionally non-tuned thalamic inputs in layer IV of striate cortex and gives rise to selectivity to more complex features along the cortical visual hierarchy. On the contrary, in the whisker-mediated touch system, directional selectivity exists in the peripheral sensory afferents innervating vibrissae follicles and gets weaker along the ascending whisker-to-barrel pathway. Thirdly, in contrast to visual system where the arrangement of neurons across the cortical surface forms a precise “pinwheel”-like orientation preference topographic map (Hubel and Wiesel, 1974; Blasdel and Salama, 1986; Grinvald et al., 1986; Bonhoeffer and Grinvald, 1991; Ohki et al., 2005, 2006), the evidence on a topographic directional tuning map in barrel field of SI is weak and controversial in the literature. While directional preference mapping was observed in VPM (Timofeeva et al., 2003), neurons in layer IV barrels exhibit weak direction preference map (Bruno et al., 2003; Andermann and Moore, 2006). Weak correlation between the angular tuning and position of neurons with respect to the center of barrel column was observed in layer II/III of adult rats through tetrode recording (Andermann and Moore, 2006) as well as two-photon calcium imaging (Kremer et al., 2011). However, such an angular preference spatial mapping is absent in supra-granular layers in juvenile rats (Kerr et al., 2007). In layer II/III, two-photon imaging revealed orientation-specific responses were organized in a locally heterogeneous and spatially distributed manner (Kwon et al., 2018). Additionally, neurons with similar orientation preference exhibited higher correlation in their trial-to-trial response variability.

Although it has been shown that rats are capable of discriminating between different orientations of an object using all of their whiskers (Polley et al., 2005), direction selectivity of single cortical units may or may not contribute to this discrimination. Difference in the kinematics of the contact of multiple whiskers along with the ego head motions could provide the information about the orientation of an object. Thus, the extent to which rats can extract the direction of a vibro-tactile stimulus using only one whisker is not yet known. Recent findings revealed that mice learned to detect optical micro-stimulation of a sparse group of supra-granular neurons in SI (Huber et al., 2007), as well as the difference between temporal patterns of electrical micro-stimulation (Yang and Zador, 2012). As vibrations with different orientation elicit responses in distinct populations of cortical neurons, the rat might be able to use that population information to decode orientation. A key test is to see if rats generalize the learned behavior when stimulus is presented to another whisker.

### 3.5. Linking Cortical Function and Behavioral Context

A given sensory stimulus may convey different meanings depending on the time and context of its occurrence, requiring the organism to take different courses of action. Sensory processing also changes with behavioral context: for example, high amplitude oscillations (known as mu rhythm) are observed in sensorimotor areas when subjects are immobile with focused attention (Kuhlman, 1978; Rougeul et al., 1979; Bouyer et al., 1981). Similar oscillations were observed in membrane potentials recorded from layer II/III neurons of mice SI in receptive mode (Crochet and Petersen, 2006). In generative mode during free whisking, however, the synchronous fluctuations were suppressed and decorrelated (Crochet and Petersen, 2006; Poulet and Petersen, 2008; Gentet et al., 2010, 2012). Beyond the spontaneous oscillations, sensory stimuli delivered to whiskers of awake rats and mice evoked a smaller response amplitude in the generative mode compared to receptive mode (Castro-Alamancos, 2004; Ferezou et al., 2006, 2007). Similar response suppression during active behavior was observed in rat auditory cortex (Otazu et al., 2009), while a response enhancement was observed in visual cortex (Niell and Stryker, 2010; Keller et al., 2012). Functional interaction between sensory and motor areas at different behavioral modes (Matyas et al., 2010; Niell and Stryker, 2010; Keller et al., 2012) and thalamocortical synaptic depression (Castro-Alamancos and Oldford, 2002; Otazu et al., 2009; Poulet et al., 2012) could account for changes in the sensory-driven response dynamics during active behavior. Grion et al. (2016) reported increased hippocampal theta band oscillations during texture discrimination task compared to a memory task in rats. This was accompanied by an enhanced phase-lock synchronization between whisking rhythm, SI neuronal spiking activity and hippocampal theta oscillation. Future paired recordings from primary somatosensory cortex and primary motor cortex or sensorimotor thalamic areas in awake rodents are required to understand the functional role and interaction of these areas in sensory processing and sensation.

Cortical neurons process information on a background of ongoing activity with distinct spatiotemporal dynamics forming various cortical states. During wakefulness, cortical state changes constantly in relation to behavioral context, attentional level or general motor activity. A common observation in awake rodents is the rapid change in spontaneous cortical activity from high-amplitude, low-frequency fluctuations referred to as synchronized state (e.g., when animals are quiet), to faster and smaller fluctuations, referred to as desynchronized state (e.g., when animals are active). Fazlali et al. (2016) recently showed this re-organization of the activity of cortical networks strongly affects sensory processing. In the desynchronized state, cortical neurons showed lower stimulus detection threshold, higher response fidelity, and shorter response latency with a prominent enhanced late response. Interestingly, changes in the activity of a small population of locus coeruleus (LC) neurons preceded and predicted the changes in the cortical state: the cross-correlation of the LC firing profile with the cortical state was maximal at an average lag of -1.2 s.

### 3.6. Link to Perception

It is not clear how and where in neocortex the perception of the tactile information emerges. However, a prime candidate for perceptual judgments and navigation based on tactile information is the prefrontal cortex (PFC). Somatosensory cortex projects into the dorsal part of medial prefrontal cortex (mPFC) (Conde et al., 1995)—homolog of primate dorsolateral prefrontal cortex. There are several lines of evidence indicating that in rats, mPFC and in particular its dorsal bank is involved in memory and delayed tasks (Larsen and Divac, 1978; Thomas and Brito, 1980; Eichenbaum et al., 1983; Wolf et al., 1987; Brabander et al., 1991; Granon et al., 1994; Verma and Moghaddam, 1996, but see de Bruin et al., 1994; Sánchez-Santed et al., 1997; Ragozzino et al., 1998). Prefrontal cortex also projects to hippocampus both directly and indirectly through lateral entorhinal cortex. The entorhinal cortex gates sensory information to hippocampus and its lesioning impairs spatial representation (Brun et al., 2008). Moreover, population dynamics of place-selective grid cells in the medial entorhinal cortex predict adaptive hippocampal remapping (Fyhn et al., 2007). Somatosensory cortex projects to the lateral entorhinal cortex through indirect projections via perirhinal cortex and also via weaker direct projections. This potentially forms an additional pathway of vibrissal information to hippocampus.

## 4. Concluding Remarks

Recent years have witnessed a revitalization of interest in rodent models not only in systems neuroscience, but also in the whole body of neuroscience research. This revitalization is partly due to availability of an increasingly powerful array of experimental approaches from optogenetics and two-photon imaging to whole-cell and intracellular electrophysiology and labeling that are challenging to apply to their full potential in primates. Availability of a broad range of genetically modified mouse lines offer scientists the tools to precisely target neuronal circuits and specific cell-types to study their function. The flat surface of the cortex in rodents without sulci and gyri along with its relatively small size is an asset for application of the state-of-the-art battery of techniques in observation and perturbation of neuronal activity. While the rodent somatosensory cortex is probably the most studied system in the literature, providing an immense amount of data from genome expression to cell types and neuronal circuitry, yet there is a huge gap in our understanding and knowledge about how this system functions. Filling this gap requires a comprehensive and coordinated drive from multiple disciplines including but not limited to cellular, systems, computational, behavioral and cognitive neuroscience.

The somatosensory system is an expert system in rodents. This system comprises one of the major channels through which rodents as nocturnal animals collect information about their surrounding environment, making this system an ideal model system to understand the neuronal computations and their underlying cellular and neuronal mechanisms in information processing and decision making. Recent studies reveal complex cognitive functions in rodent somatosensation previously reported in humans and primates such as evidence accumulation for optimal decision making and forming abstract concepts of noisy stimulation patterns (Fassihi et al., 2014; Zuo and Diamond, 2019b). Yet, further behavioral studies are required to unveil the cognitive abilities in rodents. The role of different connections and areas in this system (see Figure 1) such as vSII, vMI, TRN, ZI, and SC in different contextual and behavioral conditions is yet to be understood. Within cortical areas, the effect of different laminae and a variety of cell types (Narayanan et al., 2017) within this architecture on different aspects of sensory processing and behavior is not clear, and requires further investigation in future studies.

## Author Contributions

MA drafted and wrote the manuscript.

### Conflict of Interest Statement

The author declares that the research was conducted in the absence of any commercial or financial relationships that could be construed as a potential conflict of interest.
